# Evidence for association between Disrupted-in-schizophrenia 1 (*DISC1*) gene polymorphisms and autism in Chinese Han population: a family-based association study

**DOI:** 10.1186/1744-9081-7-14

**Published:** 2011-05-15

**Authors:** Fanfan Zheng, Lifang Wang, Meixiang Jia, Weihua Yue, Yan Ruan, Tianlan Lu, Jing Liu, Jun Li, Dai Zhang

**Affiliations:** 1 Key Laboratory for Mental Health, Ministry of Health, Beijing, P.R. China; 2 Institute of Mental Health, Peking University, Beijing, P. R. China

**Keywords:** *DISC1*, autism, SNP, FBAT, association study

## Abstract

**Background:**

Disrupted-in-Schizophrenia 1 (*DISC1*) gene is one of the most promising candidate genes for major mental disorders. In a previous study, a Finnish group demonstrated that *DISC1 *polymorphisms were associated with autism and Asperger syndrome. However, the results were not replicated in Korean population. To determine whether *DISC1 *is associated with autism in Chinese Han population, we performed a family-based association study between *DISC1 *polymorphisms and autism.

**Methods:**

We genotyped seven tag single nucleotide polymorphisms (SNPs) in *DISC1*, spanning 338 kb, in 367 autism trios (singleton and their biological parents) including 1,101 individuals. Single SNP association and haplotype association analysis were performed using the family-based association test (FBAT) and Haploview software.

**Results:**

We found three SNPs showed significant associations with autism (rs4366301: G > C, Z = 2.872, *p *= 0.004; rs11585959: T > C, Z = 2.199, *p *= 0.028; rs6668845: A > G, Z = 2.326, *p *= 0.02). After the Bonferroni correction, SNP rs4366301, which located in the first intron of *DISC1*, remained significant. When haplotype were constructed with two-markers, three haplotypes displayed significant association with autism. These results were still significant after using the permutation method to obtain empirical *p *values.

**Conclusions:**

Our study provided evidence that the *DISC1 *may be the susceptibility gene of autism. It suggested *DISC1 *might play a role in the pathogenesis of autism.

## Background

Autism is a severe neurodevelopmental disorder mainly characterized by impairment in social interaction, communicative deficits, and repetitive and stereotyped patterns of behaviors or interests [1]. Since autism was first described as a disorder by Dr. Leo Kanner in 1943 [2], the speculation about its etiology has become an intriguing field which attracts a large number of scientists. To date, compelling evidence from twin and family studies has indicated a strong genetic involvement in the etiology of autism and the estimated heritability is over 90% [3-7]. However, the genetic etiology remains elusive.

Several lines of evidence from postmortem [8-10] and structural magnetic resonance imaging (MRI) [11-16] supported the existence of brain abnormality in autism. It indicated that brain abnormalities in autism are not limited to a single brain area but involve different structures within a globally affected neuronal network. Furthermore, functional MRI studies in autism patients have indicated that alterations in task related connectivity, including enhanced activation and connectivity in posterior areas, enhanced reliance on visuospatial abilities for verbal and visual reasoning and reduced frontal systems connectivity [17-21]. All together, these previous studies suggested that abnormalities of neurodevelopment might be the etiology of autism. Considering the heritability of autism is relatively high, genes which play important roles in neurodevelopment might be candidate genes for autism.

Disrupted-in-Schizophrenia 1 (*DISC1*) is a candidate gene of autism, which has been demonstrated to involve in neuronal migration [22-24], neurite outgrowth [25,26] and axon targeting [27] during brain development. The *DISC1*, on chromosome 1q42, was originally identified from the breakpoint of a hereditary chromosomal translocation in a large Scottish pedigree [28-30]. It has 13 exons spanning over 410 kb and encodes a cytosolic scaffold protein with coiled-coil-rich C-terminus which interacts with multiple proteins involved in various functions. As one of the most interesting candidate genes for major mental illness, *DISC1 *have been demonstrated to associate with schizophrenia, depression, bipolar disorder and schizoaffective disorder in several independent populations by multiple association studies [31-37]. Meanwhile, investigations into function of *DISC1 *have revealed that it may contribute to risk for psychiatric disorders through its effects on the processes of neurodevelopment [25,38-43]. Recently, Niwa *et al. *reported that transient knockdown of *Disc1 *during embryo development of mice resulted in selective abnormalities in postnatal mesocortical dopaminergic maturation and adult behavioral deficits [44].

Autism and schizophrenia share neurocognitive defects such as impaired executive function and social functioning [45,46]. Thus, the impairment of neurodevelopment may be the common underlying mechanism of these two disorders. Recent studies have showed a genetic overlap between these two disorders [47,48]. Therefore, *DISC1 *was selected as a candidate gene for autism. Two reports have revealed some abnormalities of *DISC1 *in three individuals with autism spectrum disorders (ASD) [49,50]. Moreover, a Finnish group has represented a significant association of *DISC1 *with ASD. They established association between autism and a *DISC1 *intragenic microsatellite D1S2709 and found that one intragenic SNP, rs1322784, was associated with Asperger syndrome [51]. Their study indicated that *DISC1 *might also play a role in the etiology of autism. However, the results of association studies were not consistent. In another association study, no significant association of *DISC1 *polymorphism with ASD was found in Korean population [52].

Considering the biological functions of DISC1 protein and the positive results of previous association studies between *DISC1 *and mental disorders, we hypothesized that *DISC1 *might be involved in the etiology of autism. To explore whether *DISC1 *is the susceptibility gene of autism in Chinese Han population, we performed this family-based association study to identify the association between *DISC1 *polymorphisms and autism.

## Materials and methods

### Subjects

Three hundred and sixty seven Chinese Han family trios (singleton autistic disorder patients and their unaffected biological parents) were recruited for the present study at the Institute of Mental Health, Peking University, China. Of the 367 autistic child probands, 336 were male and 31 were female. The mean age of the children at the time of testing was 7.5 years (ranged from 3 to 17 years). Diagnoses of autism were established by two senior psychiatrists. All patients fulfilled the DSM-IV (Diagnostic and Statistical Manual of Mental Disorders, Fourth Edition) criteria for autistic disorder. To assess the cases, childhood autism rating scale (CARS) [53] and autism behavior checklist (ABC) [54] were used. Children with fragile X syndrome, tuberous sclerosis, a previously identified chromosomal abnormality, or any other neurological condition suspected to be associated with autism were excluded. All subjects provided written informed consent signed by their legal guardians (i.e., their parents) for participation in this study. This study was approved by the Ethics Committee of the Institute of Mental Health, Peking University.

### Genotyping

**Figure 1:**
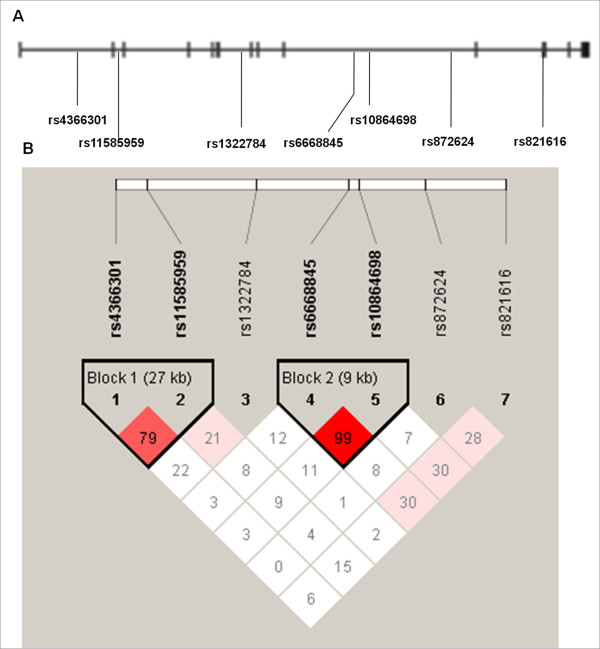
***DISC1 *gene locus and linkage disequilibrium (LD) structure**. **(A) **A diagram showing the exonic structure of *DISC1 *gene (black). The single nucleotide polymorphisms (SNPs) used in this study are shown in relation to the exons. **(B)**The linkage disequilibrium (LD) structure of the *DISC1 *region in the total autism samples according to Haploview (solid spine of LD, *D'*> 0.7). Markers with LD (*D' *< 1 and LOD > 2) are shown in red through pink (color intensity decreases with decreasing D' value). Regions of low LD (*D' *< 1 and LOD < 2) are shown in white. Two blocks were generated by Haploview.

Genomic DNA was extracted from the blood using a Qiagen QIAamp DNA Mini Kit. We selected seven single nucleotide polymorphisms (SNPs) in the *DISC1 *gene according to the dbSNP http://www.ncbi.nlm.nih.gov/SNP/ and the HapMap phase Ⅱ Chinese Han in Beijing (CHB) genotype dataset http://hapmap.ncbi.nlm.nih.gov/, including rs4366301, rs11585959, rs1322784, rs6668845, rs10864698, rs872624 in introns and rs821616 in exon (Figure 1). SNPs with minor allele frequency (MAF) >0.05 were selected and pair-wise tagging in the Tagger module implemented in Haploview v4.1 program was used to select SNPs that could capture >80% of the markers with *r*^*2*^>0.8. Meanwhile, the physical position of SNPs and the previous positive results were considered too.

Direct DNA sequencing was used for analyzing rs11585959. The other six SNPs (rs4366301, rs1322784, rs6668845, rs10864698, rs872624 and rs821616) were analyzed by polymerase chain reaction-restriction fragment length polymorphism (PCR-RFLP) analysis. The information of primers and PCR-RFLP analysis is given in Table 1. The PCR amplification was performed in a 25 μl volume containing GC Buffer (TaKaRa), 200 μM of each dNTPs, 0.3 μM of each primer, 1 U of Taq DNA polymerase, and 40 ng of the genomic DNA. The conditions used for PCR amplification were an initial denaturation phase at 94°C for 5min, followed by 38 cycles at 94°C for 30 sec, annealing at 53-65°C for 30-45 sec, and extension at 72°C for 30 sec, followed by a final extension phase at 72°C for 7 min. A 15 μL aliquot of the PCR product mixtures was completely digested with 4 units of restriction enzyme overnight. Digestion products were visualized through ethidium bromide staining after electrophoresis in 1.5%-2% agarose gels. The DNA sequencing was performed after cleaning the PCR product using a BigDye Terminator Cycle Sequencing Ready Reaction Kit with Ampli Taq DNA polymerase (PE Biosystem). The inner primers were used for the cycle-sequencing reaction, and the fragments were separated by electrophoresis on an ABI PRISM 377-96 DNA Sequencer (Applied Biosystem, Foster City, U.S.A).

**Table 1:** Information of the primers and PCR-RFLP Analysis of seven SNPs in *DISC1 *gene

SNP	Position	Primer sequence (5'-3')	Product (bp)	RFLP	Allele (bp)	
rs4366301	Intron 1	F: AGAAGACTAGGAAAAATAACT R: ATAAACACTGAACAGAATGTC	406	ScrFⅠ	C: (43/265/98)	G: (43/363)
rs11585959^a^	Intron 2	F: TGACATTCTACCTTCTCTCTC R: ACTACCTTTATTACCATCTTC	556	-	-	-
rs1322784	Intron 6	F: CCTCCTCTGTTGAAAGTAGGT R:GGAAGAAAGTCTGAATGTGAC	645	Van91Ⅰ	A: (201/444)	G: (645)
rs6668845	Intron 9	F:AAAAAAAAAAAATCAACTGAG R:CCCAAATCTTTCATAGTGACT	572	TaiⅠ	G: (442/130)	A: (572)
rs10864698	Intron 9	F: TCCAGAGCCAGTGAAATGTTC R: TTGTGCCTGAATGAATGAGAC	630	MunⅠ	A: (381/249)	G: (630)
rs872624	Intron 9	F: ACAAAACCAGAAACCTTGAGT R: ACATATTAGGGAAACTGAATG	757	NdeⅠ	G: (506/251)	A: (757)
rs821616	Exon 11	F: GTATTGGGCTGCTGAGTCTG R: GACCTCTTTCTGTTCACCTCC	540	BsrⅠ	T: (204/336)	A: (540)

PCR-RFLP, polymerase chain reaction-restriction fragment length polymorphism; SNP, single nucleotide polymorphism; F, forward; R, reverse.

^a^direct sequencing was applied for rs11585959.

### Statistical Analyses

Prior to analysis, Mendelian inconsistencies were checked using the PEDCHECK program, version 1.1 [55]. Deviations in the genotype counts from the Hardy-Weinberg equilibrium were tested using a chi-square goodness-of-fit test. The pairwise linkage disequilibrium (LD) analysis was applied to detect the inter-marker relationship with Haploview http://www.broad.mit.edu/mpg/haploview/, using *D' *values. The family-based association test (FBAT) was performed with FBAT program v1.5.1 [56]. The FBAT program uses generalized score statistics to perform a variety of transmission disequilibrium tests (TDT), including haplotype analyses. Moreover, the FBAT program provides estimates of haplotype frequencies and pairwise linkage disequilibrium (LD) between the specified markers http://www.biostat.harvard.edu/~fbat/default.html. The global haplotype tests of association were performed under "multiallelic" mode in haplotype FBAT. Meanwhile, the individual haplotype tests were conducted under "biallelic" mode in haplotype FBAT. Family-based association tests were performed under an additive model in the present study. The significance level for all statistical tests was two-tailed (*p *< 0.05). We also performed the association analysis by Haploview version 4.1 http://www.broad.mit.edu/mpg/haploview[57].

## Results

We tested a total of seven SNPs over the 338 kb region of 1q42 with *DISC1 *in 367 Chinese Han autism trios. All seven SNPs were polymorphic with minor allele frequency (MAF) >5% and were then used as genetic markers for the association study. A number of sample genotypes could not be assigned due to repeated PCR failure or unclear genotype results, including 10 genotypes for rs4366301 and rs11585959; 5 for rs1322784; 9 for rs6668845; 7 for rs10864698; 30 for rs872624 and 3 for rs821616. None of the genotype distributions of these SNPs in patients significantly deviated from Hardy-Weinberg equilibrium (*p *> 0.05, data not shown). Allele frequencies and the results of FBAT for single SNP analysis are shown in Table 2. Univariate (single-marker) FBAT demonstrated that variant alleles at three SNPs showed a preferential transmission (rs4366301 G: Z = 2.872, *p *= 0.004; rs11585959 T: Z = 2.199, *p *= 0.028; rs6668845 A: Z = 2.326, *p *= 0.020) (Table 2). Moreover, rs4366301 remained significant with autism after the Bonferroni correction (significantly corrected *p *< 0.007, i.e. *α *= 0.05/n) which was considered as a conservative correction method. The LD patterns of the SNPs were measured with the solid spine of LD option of Haploview (*D'*>0.7). Two LD blocks were identified (Figure 1). To determine whether any specific haplotype would confer a higher risk for autism, the specific and global-haplotype FBAT tests of association were performed (Table 3). Three haplotypes displayed significant association. The haplotype G-T (rs4366301-rs11585959) revealed significant excess transmission from parents to affected offspring both in the specific and global haplotype FBAT (*p *= 0.0015 and 0.0062, respectively). In addition, haplotype constructed from the A allele of rs6668845 and the G allele of rs10864698 demonstrated an excess transmission (*p *= 0.024 and 0.0064, respectively) and haplotype constructed from the G allele of rs6668845 and the A allele of rs10864698 displayed an undertransmission (*p *= 0.0499 and 0.0064, respectively). These results were still significant, after using the permutation method to obtain empirical *p *values.

**Table 2:** Results of FBAT for the seven SNPs in *DISC1 *gene

Markers	Afreq	Families	S	E(s)	Z	*p*-values
**rs4366301**	C:0.831 G:0.169	169 169	213.00 125.00	233.00 105.00	-2.872 2.872	**0.004** **0.004**
**rs11585959**	C:0.243 T:0.757	216 216	123.00 309.00	141.00 291.00	-2.199 2.199	**0.028** **0.028**
**rs1322784**	G:0.369 A:0.631	242 242	206.00 278.00	202.50 281.50	-0.392 0.392	0.695 0.695
**rs6668845**	G:0.332 A:0.668	244 244	172.00 316.00	193.00 295.00	-2.326 2.326	**0.020** **0.020**
**rs10864698**	A:0.330 G:0.670	242 242	175.00 309.00	191.50 292.50	-1.831 1.831	0.067 0.067
**rs872624**	G:0.345 A:0.655	232 232	168.00 296.00	180.00 284.00	-1.372 1.372	0.170 0.170
**rs821616**	A:0.881 T:0.119	130 130	186.00 74.00	181.50 78.50	0.728 -0.728	0.467 0.467

FBAT, family-based association test; Afreq, allelic frequency; Families, number of informative families;

S, test statistics for the observed number of transmitted alleles; E(S), expected value of S under the null hypothesis (i.e., no linkage or association).

Significant *p *values (*p *< 0.05) are in boldface.

**Table 3:** Results of Haplotype association analysis for the SNPs with linkage disequilibrium

Markers	Haplotype	Freq	Z	*p*	Global Haplotype test		***p***^***a***^
						
					**χ**^**2**^	*p*	
rs4366301-rs11585959	G-T	0.160	3.166	0.0015	12.362	0.0062	0.004
rs6668845-rs10864698	A-G	0.665	2.257	0.0240	10.095	0.0064	0.026
rs6668845-rs10864698	G-A	0.327	-1.960	0.0499	10.095	0.0064	0.026

Freq, frequency of the haplotype. ^*a *^*p *values using permutation test. Global haplotype represents the haplotype using all possible variants.

We also performed the single SNP association and haplotype analysis with Haploview, the results were similar to those using FBAT method. (Additional file 1, Table S1, Table S2).

## Discussion

*DISC1 *is one of the most promising susceptibility genes for major mental disorders. Genetic studies had indicated that *DISC1 *was a susceptibility gene for schizophrenia, depression, bipolar disorder and schizoaffective disorder [31-37]. Many investigators have considered the possibility that adult brain function and behavior are influenced by neuronal network formation during early development [58-61]. Considering the established biological functions of the DISC1 protein and the structural and functional abnormalities in brains of autism patients, *DISC1 *was a candidate gene for autism. Up to know, besides two case reports, only one published research detected *DISC1 *was associated with autism and Asperger syndrome using family-based association analysis. However, the results did not replicated in a Korean population. To investigate *DISC1 *as potential autism susceptibility gene, we performed a family-based association study in Chinese Han population. In the present study, we analyzed seven tag SNPs in *DISC1 *for association with autism in 367 Chinese Han family trios. The results of our study demonstrated that three SNPs, rs4366301, rs11585959, and rs6668845 were associated with autism (Table 2). Among these three SNPs, rs4366301 had the most significance (*p *= 0.004). Even after Bonferroni correction, rs4366301 was still significant. When haplotypes were constructed with two markers, three haplotypes displayed positive association. The results remained significant when the global haplotype FBAT was performed (Table 3). Our research established the association between autism and the *DISC1 *gene.

In a previous study, a Finnish group reported a positive association of rs1322784 with Asperger syndrome besides a significant association of a *DISC1 *intragenic microsatellite with autism [51]. However, we didn't replicate this positive finding in 367 autism trios in Chinese Han population. No significant association was obtained for rs1322784 in our study (*p *= 0.695). One reason is that our research mainly focused on the genetic research of autism, while the Finnish study founded that rs1322784 was associated with Asperger syndrome. Although autism and Asperger syndrome are included in autism spectrum disorder (ASD), these two disorders have different clinical features. Therefore, the genetic etiology of autism and Asperger syndrome may be not consistent. Second, as the authors mentioned in their paper, the sample size of the Finnish study was relatively small [51]. The result should be replicated in an expanded sample. Ethnic genetic heterogeneity might be another reason for these discordant findings. In our study, major allele frequency for rs1322784 in autism cases was 0.617 compare to 0.83 in Finnish population (Additional file 1, Table S3)[51,52]. The allele frequency for rs1322784 varies in different population as shown in the data from the International HapMap project. In CEU (Utah residents with Northern and Western European ancestry from the CEPH collection) population the major allele frequency (MAF) of rs1322784 is 0.786, as well as 0.589 in Chinese Han population in Beijing (CHB). Though our study did not replicate the association between rs1322784 and autism in Chinese Han population, we observed strong association of rs4366301 in *DISC1 *with autism.

In the Finnish study, Kilpinen *et al. *detected suggestive association with haplotype HEP3 (rs751229 and rs3738401) [62] in the Asperger syndrome study sample using TRANSMIT software. However, this haplotype was not associated with Asperger syndrome and autism when analysis was performed by FBAT software. Moreover, single SNP association analysis of rs751229 and rs3738401 obtained no significant association with Asperger syndrome and autism [51]. We constructed the LD patterns between rs751229 and rs3738401 in the Haploview by using the genotyping date (only SNPs with MAF > 0.05) from the HapMap project http://www.hapmap.org. In Chinese Han population, genotyping date of rs751229 was not available in HapMap project. However, SNP rs2082552 and rs823165 were genotyped. The positions of these two SNPs were very close to rs751229 (the distance is 649 bp and 907 bp, respectively). LD analysis demonstrated that rs3728401, rs2082552, and rs823165 were not located within one linkage disequilibrium block (Additional file 1, Figure S1). Pairwise *D*' between rs3728401 and rs2082552 is 0.64, while *D' *between rs3728401 and rs823165 is 0.66. These results suggested linkage disequilibrim of rs3738401 and rs751229 might not exist in Chinese Han population. Therefore, we did not perform the association study of these two SNPs with autism in Chinese Han population. In our study, we detected two other haplotypes which were associated with autism. The haplotype G-T (rs4366301-rs11585959) and A-G (rs6668845-rs10864698) demonstrate significant excess transmission from parents to affected offspring.

Many association studies have replicated a positive association of rs821616 which is a missense polymorphism leading to a serine-to-cysteine substitution at amino acid 704 (Ser704Cys)in *DISC1 *with schizophrenia [32,36,37]. Our group have also demonstrated the association between rs821616 and schizophrenia in Chinese Han population[32]. Function studies have reported that the substitution induced by rs821616 polymorphism may affect cognition [37,63] and the underlying mechanism may be an altered interaction of DISC1 with its binding partners or disruption of its signaling pathways [64,65]. Therefore, we genotyped this SNP in our study However, we obtained no significant support for the association of rs821616 with autism (*p *= 0.467). This result is consistent with the results of the previous studies in Korean population [52] and Finnish population [51]. It indicated that rs821616 may not involve in the etiology of autism. However, we found that other SNPs were significantly associated with autism in Chinese Han population.

DISC1 is a pleiotropic protein with many purported functions, subcellular locations, binding partners and plays roles in many aspects of neurodevelopment [22,39,66-69]. The middle part of DISC1 had been identified as a DISC1 self-association domain [23]. In contrast, the C-termianl part of DISC1 binds to important interacting molecules.. Interactions with LIS1, NDEL, FEZ1, integrin and other proteins indicated that many of the proteins of the "DISC1 network" are involved in pathways critical for neuronal polarization and migration, formation of synapses, and dendritic growth and branching. Up to now, researches have demonstrated that *DISC1 *is a susceptibility gene for mental disorders such as autism, schizophrenia and bipolar disorder. However, it is possible that the underlying pathogeneses of *DISC1 *for different psychiatric disorders might be different.

In conclusion, the present study demonstrated a positive association of *DISC1 *with autism in Chinese Han population. Our results provided evidence that *DISC1 *might involve in the etiology of autism. Further researches of association analysis across populations are needed.

## Competing interests

The authors declare that they have no competing interests.

## Authors' contributions

FZ and LW contributed equally to this work.

FZ and LW designed the study and drafted the manuscript. FZ performed the experiment, analyzed the data and interpreted the results. MJ, WY, YR, TL, JL and JL participated in data collection. WY and DZ supervised the study. All authors read and approved the final manuscript.

## Supplementary Material

Additional file 1 **1-DISC1 and autism**. **Table S1**. Results of single marker association for seven SNPs in *DISC1 *gene in 367 trios by Haploview. **Table S2**. Results of Haplotype association analysis for the SNPs with linkage disequilibrium in 367 trios by Haploview. **Table S3**. Frequencies of different alleles and genotypes in different association studies **Figure S1**. *DISC1 *gene linkage disequilibrium (LD) structure in Chinese Han in Beijing (CHB) using HapMap date by Haploview. Tables and figures Click here for file
