# Supplementary material for: Evidence for association between Disrupted-in-schizophrenia 1 (DISC1) gene polymorphisms and autism in Chinese Han population: a family-based association study
Source: Behav Brain Funct. 2011 May 15;7:14. doi: 10.1186/1744-9081-7-14 (PMC3113723; doi:10.1186/1744-9081-7-14)
Supplement: Additional file 1 — 1-DISC1 and autism. Table S1. Results of single marker association for seven SNPs in DISC1 gene in 367 trios by Haploview. Table S2. Results of Haplotype association analysis for the SNPs with linkage disequilibrium in 367 trios by Haploview. Table S3. Frequencies of different alleles and genotypes in different association studies Figure S1. DISC1 gene linkage disequilibrium (LD) structure in Chinese Han in Beijing (CHB) using HapMap date by Haploview. Tables and figures [file 1744-9081-7-14-S1.DOC]

**Additional file 1**

**Table S1.** Results of single marker association for seven SNPs in *DISC1* gene in 367 trios by Haploview.

| Marker | Overtransmitted | T:U | Chi Square | *p* |
| --- | --- | --- | --- | --- |
| rs4366301 | G | 117:77 | 8.247 | 0.004 |
| rs11585959 | T | 152:116 | 4.836 | 0.028 |
| rs1322784 | C | 163:156 | 0.154 | 0.695 |
| rs6668845 | A | 184:142 | 5.411 | 0.020 |
| rs10864698 | G | 179:146 | 3.351 | 0.067 |
| rs872624 | A | 165:141 | 1.882 | 0.170 |
| rs821616 | A | 81:72 | 0.529 | 0.467 |

Overtransmitted is the allele overtransmitted to affected offspring;

T:U is the ratio of transmissions to non-transmissions of the overtransmitted allele.

**Table S2.** Results of Haplotype association analysis for the SNPs with linkage disequilibrium in 367 trios by Haploview

| Markers | Allele | Freq | T:U | Chi Square | *p* |
| --- | --- | --- | --- | --- | --- |
| rs4366301-rs11585959 | G-T | 0.162 | 117.1:74.0 | 9.694 | 0.002 |
| rs6668845-rs10864698 | A-G | 0.667 | 184.5:142.5 | 5.398 | 0.020 |
|  | G-A | 0.330 | 144.5:182.5 | 4.41 | 0.036 |

Freq: the population frequency for this haplotype;

T:U is the ratio of transmissions to non transmissions of the haplotype to affected offspring.

**Table S3.** Frequencies of different alleles and genotypes in different association studies

| Marker | Allele | Finnish family-based study | | | Korean Case-control study | | | | Chinese Family-based study | |
| --- | --- | --- | --- | --- | --- | --- | --- | --- | --- | --- |
|  |  | Major allele frequency | | *p* | genotype | Genotypic distributions | | *p* | Major  allele freqency | *p* |
|  |  | AUT cases | AS cases |  |  | ASD | controls |  | AUT cases |  |
| rs1630250 | **G**/C | 0.73 | 0.72 | >0.05 | – | – | – | – | – | – |
| rs1655285 | **G**/C | 0.93 | 0.96 | >0.05 | – | – | – | – | – | – |
| D1S251 |  |  |  | >0.05 | – | – | – | – | – | – |
| rs751229 | **T**/C | 0.56 | 0.58 | >0.05 | – | – | – | – | – | – |
| rs3738401 | **G**/A | 0.74 | 0.72 | >0.05 | AA  AG  GG | 0.036  0.405  0.559 | 0.043  0.393  0.564 | >0.05 | – | – |
| rs1322784 | **A**/G | 0.83 | 0.83 | **0.0195**^a^ | – | – | – | – | 0.617 | >0.05 |
| rs967244 | **A**/G | 0.82 | 0.81 | >0.05 | – | – | – | – | – | – |
| rs6675281 | **C**/T | 0.89 | 0.89 | >0.05 | – | – | – | – | – | – |
| rs1000731 | **C**/T | 0.64 | 0.68 | >0.05 | – | – | – | – | – | – |
| D1S2709 |  |  |  | **0.01**^b^ | – | – | – | – | – | – |
| rs821616 | **A**/T | 0.63 | 0.65 | >0.05 | AA  AT  TT | 0.001  0.221  0.769 | 0.013  0.184  0.803 | >0.05 | 0.881 | >0.05 |
| rs1411771 | **T**/C | 0.71 | 0.67 | >0.05 | – | – | – | – | – | – |
| rs980989 | **G**/T | 0.79 | 0.73 | >0.05 | – | – | – | – | – | – |
| rs3738402 | **C**/T | – | – | – | CC  CT  TT | 0.626  0.297  0.077 | 0.633  0.311  0.056 | >0.05 | – | – |
| rs4366301 | **C**/G | – | – | – | – | – | – | – | 0.802 | **0.004** |
| rs11585959 | **T**/C | – | – | – | – | – | – | – | 0.779 | **0.028** |
| rs6668845 | **A**/G | – | – | – | – | – | – | – | 0.698 | **0.020** |
| rs10864698 | **G**/A | – | – | – | – | – | – | – | 0.690 | >0.05 |
| rs872624 | **A**/G | – | – | – | – | – | – | – | 0.666 | >0.05 |

AUT: autism; AS: asperger syndrome; ASD: autism spectrum disorder; ^a^, *p* value in asperger syndrome sample males only; ^b^, *p* value in autism sample.

**Figure S1.** *DISC1* gene linkage disequilibrium (LD) structure in Chinese Han in Beijing (CHB) using HapMap date by Haploview.


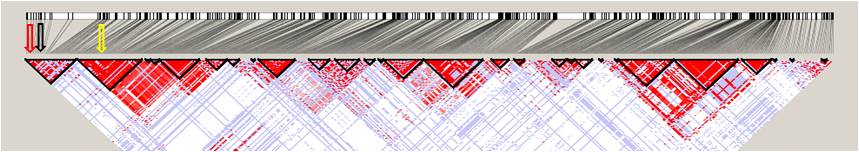


The linkage disequilibrium (LD) structure of the DISC region in the total study samples according to Haploview (solid spine of LD, *D’*> 0.8). The position of rs2082552 and rs823165 are indicated by red arrow and black arrows respecitively, whereas rs3738401 identified in this study is indicated in yellow arrow. These SNPs were in two different blocks.
